# Bis{1-[2-(diphenyl­phosphino)­ethyl]-3-ethylimidazol-2-yl­idene}palladium(II) bis­(hexa­fluoridophosphate) acetonitrile 2.85-solvate

**DOI:** 10.1107/S160053681001319X

**Published:** 2010-04-14

**Authors:** A. Ahmida Aziza, Ulrich Flörke, Hans Egold, Gerald Henkel

**Affiliations:** a Department Chemie, Fakultät für Naturwissenschaften, Universität Paderborn, Warburgerstrasse 100, 33098 Paderborn, Germany

## Abstract

In the structure of the title compound, [Pd(C_19_H_21_N_2_P)_2_](PF_6_)_2_·2.85CH_3_CN, the two six-membered NHC-phosphane chelate rings form a distorted square-planar coordination geometry around the Pd^II^ atom, which lies 0.212 (1) Å above the coordination plane. The sum of the bond angles at Pd^II^ is 358.3°, with C—Pd—P bite angles of 84.03 (10) and 83.54 (9)°. The structure includes three acetonitrile solvent mol­ecules, one with partial site occupation and one with severe disorder, which was eventually excluded from the refinement.

## Related literature

For the structures of related Pd^II^ complexes, see: Chiu *et al.* (2005 ▶); Lee *et al.* (2004*a* ▶,*b* ▶); Navarro *et al.* (2004 ▶); Tsoureas *et al.* (2003 ▶). For refinement aspects, see: Spek (2009 ▶).
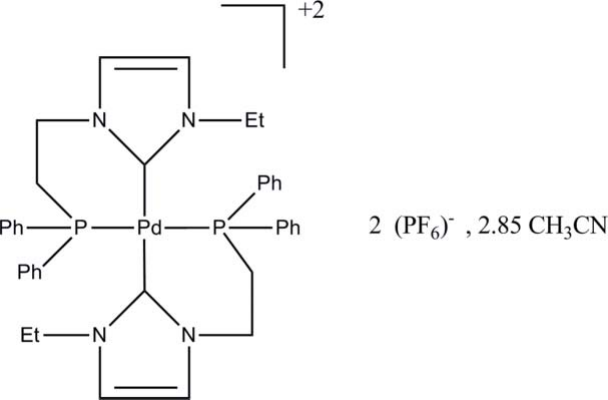


## Experimental

### 

#### Crystal data

[Pd(C_19_H_21_N_2_P)_2_](PF_6_)_2_·2.85C_2_H_3_N
*M* *_r_* = 1130.45
Triclinic, 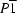

*a* = 11.114 (2) Å
*b* = 11.343 (2) Å
*c* = 20.243 (4) Å
α = 77.490 (4)°
β = 83.580 (5)°
γ = 85.301 (4)°
*V* = 2471.4 (8) Å^3^
*Z* = 2
Mo *K*α radiation
μ = 0.59 mm^−1^
*T* = 120 K
0.42 × 0.20 × 0.20 mm

#### Data collection

Bruker SMART APEX diffractometer
Absorption correction: multi-scan (*SADABS*; Bruker, 2002 ▶) *T*_min_ = 0.790, *T*_max_ = 0.891
20500 measured reflections
10802 independent reflections
9025 reflections with *I* > 2σ(*I*)
*R*_int_ = 0.032

#### Refinement

*R*[*F* ^2^ > 2σ(*F* ^2^)] = 0.047
*wR*(*F* ^2^) = 0.129
*S* = 1.05
10802 reflections
584 parameters
H-atom parameters constrained
Δρ_max_ = 0.90 e Å^−3^
Δρ_min_ = −0.56 e Å^−3^

### 

Data collection: *SMART* (Bruker, 2002 ▶); cell refinement: *SAINT* (Bruker, 2002 ▶); data reduction: *SAINT*; program(s) used to solve structure: *SHELXS97* (Sheldrick, 2008 ▶); program(s) used to refine structure: *SHELXL97* (Sheldrick, 2008 ▶); molecular graphics: *SHELXTL* (Sheldrick, 2008 ▶); software used to prepare material for publication: *SHELXTL*.

## Supplementary Material

Crystal structure: contains datablocks I, global. DOI: 10.1107/S160053681001319X/wm2316sup1.cif

Structure factors: contains datablocks I. DOI: 10.1107/S160053681001319X/wm2316Isup2.hkl

Additional supplementary materials:  crystallographic information; 3D view; checkCIF report

## Figures and Tables

**Table:** 

Pd1—C31	2.031 (3)
Pd1—C1	2.033 (3)
Pd1—P1	2.3085 (9)
Pd1—P2	2.3100 (9)

**Table:** 

C31—Pd1—C1	173.56 (13)
C31—Pd1—P1	94.24 (9)
C1—Pd1—P1	84.03 (10)
C31—Pd1—P2	83.54 (9)
C1—Pd1—P2	96.46 (10)
P1—Pd1—P2	164.44 (3)

**Fig. 1.**
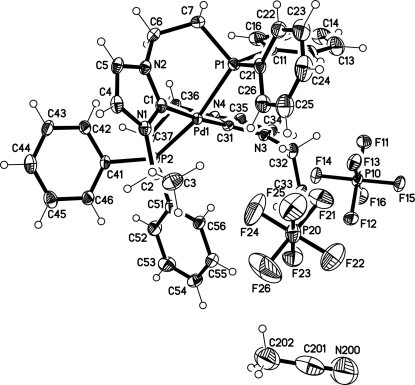
Molecular structure of the title compound. Displacement ellipsoids are drawn at the 50% probability level. For clarity, the N300 solvent molecule with partial site occupation was omitted.

## supplementary crystallographic information

### Comment

The two six-membered NHC-phosphane chelate rings form a distorted square-planar coordination geometry around the palladium(II) atom which lies 0.212 (1) Å above the coordination plane. The sum of bond angles at Pd is 358.3° with C1—Pd1—P1 and C31—Pd1—P2 bite angles of 84.03 (10)° and 83.54 (9)°, respectively. The trans C1—Pd1—C31 and P1—Pd1—P2 bond angles are 173.56 (13)° and 164.44 (3)° and thus deviate clearly from linearity. The Pd—C bond lengths (2.031 (3) and 2.033 (3) Å) are within the expected range and the Pd—P bond lengths of 2.3085 (9) and 2.3100 (9) Å also are comparable to those in other complexes containing Pd-NHC phosphane ligands (e.g. Chiu *et al.* (2005); Lee *et al.* (2004*a*,*b*); Navarro *et al.* (2004); Tsoureas *et al.* (2003)). There are no intermolecular contacts shorter than the van der Waals distances.

### Experimental

To a solution of 3-[2-(diphenylphosphino)ethyl]-1-ethylimidazolium-hexafluoridophosphate (195 mg, 0.46 mmol) in THF (10 ml) was added KN(SiMe_3_)_2_ (118 mg, 0.46 mmol + 30%) and the mixture was stirred at room temperature under N_2_ for 30 min. Then [Pd(COD)Cl_2_] (66 mg, 0.230 mmol; COD = 1,5-cyclooctadiene) was added to the reaction mixture and the colour changed from pale yellow to yellow–orange. The reaction mixture was stirred for another 2 h and then the solvent was removed under vacuum. Yellow crystals were obtained from an acetonitrile solution by diethyl ether diffusion.

### Refinement

For the final refinement, data were cut off at θ = 27.1° in order to get 99% completeness. H atoms were clearly identified in difference Fourier syntheses, idealized and refined at calculated positions riding on the C atoms with isotropic displacement parameters U_iso_(H) = 1.2U(C_eq_) or 1.5U(-CH_3_). All methyl H atoms were allowed to rotate but not to tip. There are three acetonitrile solvent molecules per asymmetric unit. Two of these could be refined easily, the N300 one with an occupation factor of 0.85 (1). It was, however, not possible to refine successfully the third heavily distorted acetonitrile molecule. After treatment of the data with the SQUEEZE facility of PLATON (Spek, 2009) refinement then proceeded smoothly.

### Crystal data

**Table:**

[Pd(C_19_H_21_N_2_P)_2_](PF_6_)_2_·2.85C_2_H_3_N	*Z* = 2
*M**_r_* = 1130.45	*F*(000) = 1150
Triclinic, *P*1	*D*_x_ = 1.519 Mg m^−^^3^
Hall symbol: -P 1	Mo *K*α radiation, λ = 0.71073 Å
*a* = 11.114 (2) Å	Cell parameters from 1002 reflections
*b* = 11.343 (2) Å	θ = 2.3–28.1°
*c* = 20.243 (4) Å	µ = 0.59 mm^−^^1^
α = 77.490 (4)°	*T* = 120 K
β = 83.580 (5)°	Prism, pale-yellow
γ = 85.301 (4)°	0.42 × 0.20 × 0.20 mm
*V* = 2471.4 (8) Å^3^	

### Data collection

**Table:**

Bruker SMART APEX diffractometer	10802 independent reflections
Radiation source: sealed tube	9025 reflections with *I* > 2σ(*I*)
graphite	*R*_int_ = 0.032
φ and ω scans	θ_max_ = 27.1°, θ_min_ = 1.8°
Absorption correction: multi-scan (*SADABS*; Bruker, 2002)	*h* = −14→14
*T*_min_ = 0.790, *T*_max_ = 0.891	*k* = −14→14
20500 measured reflections	*l* = −24→25

### Refinement

**Table:**

Refinement on *F*^2^	Primary atom site location: structure-invariant direct methods
Least-squares matrix: full	Secondary atom site location: difference Fourier map
*R*[*F*^2^ > 2σ(*F*^2^)] = 0.047	Hydrogen site location: difference Fourier map
*wR*(*F*^2^) = 0.129	H-atom parameters constrained
*S* = 1.05	*w* = 1/[σ^2^(*F*_o_^2^) + (0.0605*P*)^2^ + 3.077*P*] where *P* = (*F*_o_^2^ + 2*F*_c_^2^)/3
10802 reflections	(Δ/σ)_max_ < 0.001
584 parameters	Δρ_max_ = 0.90 e Å^−^^3^
0 restraints	Δρ_min_ = −0.56 e Å^−^^3^

### Special details

**Table:**

Geometry. All esds (except the esd in the dihedral angle between two l.s. planes) are estimated using the full covariance matrix. The cell esds are taken into account individually in the estimation of esds in distances, angles and torsion angles; correlations between esds in cell parameters are only used when they are defined by crystal symmetry. An approximate (isotropic) treatment of cell esds is used for estimating esds involving l.s. planes.
Refinement. Refinement of *F*^2^ against ALL reflections. The weighted *R*-factor wR and goodness of fit *S* are based on *F*^2^, conventional *R*-factors *R* are based on *F*, with *F* set to zero for negative *F*^2^. The threshold expression of *F*^2^ > σ(*F*^2^) is used only for calculating *R*-factors(gt) etc. and is not relevant to the choice of reflections for refinement. *R*-factors based on *F*^2^ are statistically about twice as large as those based on *F*, and *R*- factors based on ALL data will be even larger.

### Fractional atomic coordinates and isotropic or equivalent isotropic displacement parameters (Å^2^)

**Table:**

	*x*	*y*	*z*	*U*_iso_*/*U*_eq_	Occ. (<1)
Pd1	0.14297 (2)	0.58147 (2)	0.214403 (12)	0.01778 (8)	
P1	0.10291 (8)	0.69609 (8)	0.29654 (4)	0.02250 (19)	
P2	0.14709 (8)	0.50588 (8)	0.11694 (4)	0.02037 (18)	
N1	−0.0082 (3)	0.3656 (3)	0.29190 (14)	0.0242 (6)	
N2	−0.1052 (3)	0.5386 (3)	0.28212 (14)	0.0233 (6)	
N3	0.3767 (3)	0.7229 (2)	0.16884 (14)	0.0216 (6)	
N4	0.2425 (2)	0.7651 (2)	0.09788 (14)	0.0215 (6)	
C1	0.0041 (3)	0.4847 (3)	0.26744 (16)	0.0220 (7)	
C2	0.0877 (4)	0.2695 (3)	0.28886 (19)	0.0318 (8)	
H2A	0.1630	0.3058	0.2653	0.038*	
H2B	0.0633	0.2136	0.2621	0.038*	
C3	0.1126 (4)	0.1990 (4)	0.3589 (2)	0.0424 (10)	
H3A	0.1764	0.1354	0.3547	0.064*	
H3B	0.0384	0.1622	0.3822	0.064*	
H3C	0.1390	0.2537	0.3851	0.064*	
C4	−0.1260 (3)	0.3454 (3)	0.32047 (17)	0.0280 (8)	
H4A	−0.1578	0.2692	0.3406	0.034*	
C5	−0.1863 (3)	0.4538 (3)	0.31420 (17)	0.0256 (7)	
H5A	−0.2691	0.4692	0.3290	0.031*	
C6	−0.1331 (3)	0.6692 (3)	0.26246 (18)	0.0267 (7)	
H6A	−0.2208	0.6868	0.2738	0.032*	
H6B	−0.1152	0.6955	0.2126	0.032*	
C7	−0.0607 (3)	0.7417 (3)	0.2980 (2)	0.0299 (8)	
H7A	−0.0954	0.7329	0.3459	0.036*	
H7B	−0.0703	0.8284	0.2759	0.036*	
C11	0.1811 (3)	0.8350 (3)	0.2794 (2)	0.0301 (8)	
C12	0.2725 (4)	0.8492 (4)	0.3179 (2)	0.0412 (10)	
H12A	0.2886	0.7895	0.3572	0.049*	
C13	0.3400 (5)	0.9504 (5)	0.2990 (3)	0.0581 (14)	
H13A	0.4010	0.9612	0.3261	0.070*	
C14	0.3197 (5)	1.0347 (4)	0.2416 (3)	0.0620 (16)	
H14A	0.3678	1.1030	0.2285	0.074*	
C15	0.2303 (4)	1.0217 (4)	0.2027 (3)	0.0551 (14)	
H15A	0.2168	1.0810	0.1628	0.066*	
C16	0.1588 (4)	0.9219 (3)	0.2213 (2)	0.0396 (10)	
H16A	0.0960	0.9135	0.1948	0.047*	
C21	0.1273 (3)	0.6228 (3)	0.38290 (17)	0.0273 (7)	
C22	0.0872 (4)	0.6806 (4)	0.4361 (2)	0.0399 (10)	
H22A	0.0465	0.7585	0.4270	0.048*	
C23	0.1068 (5)	0.6240 (6)	0.5024 (2)	0.0529 (13)	
H23A	0.0802	0.6635	0.5387	0.063*	
C24	0.1641 (5)	0.5121 (5)	0.5151 (2)	0.0538 (13)	
H24A	0.1756	0.4733	0.5606	0.065*	
C25	0.2059 (5)	0.4536 (5)	0.4633 (2)	0.0493 (12)	
H25A	0.2468	0.3758	0.4731	0.059*	
C26	0.1879 (4)	0.5092 (4)	0.39648 (19)	0.0352 (9)	
H26A	0.2167	0.4697	0.3604	0.042*	
C31	0.2673 (3)	0.6927 (3)	0.15747 (16)	0.0200 (6)	
C32	0.4479 (3)	0.6611 (3)	0.22448 (18)	0.0294 (8)	
H32A	0.3963	0.6051	0.2581	0.035*	
H32B	0.4742	0.7216	0.2477	0.035*	
C33	0.5575 (3)	0.5912 (3)	0.1988 (2)	0.0334 (8)	
H33A	0.6026	0.5504	0.2371	0.050*	
H33B	0.6096	0.6468	0.1664	0.050*	
H33C	0.5315	0.5308	0.1761	0.050*	
C34	0.4190 (3)	0.8157 (3)	0.11711 (18)	0.0263 (7)	
H34A	0.4934	0.8534	0.1139	0.032*	
C35	0.3350 (3)	0.8424 (3)	0.07238 (18)	0.0257 (7)	
H35A	0.3384	0.9023	0.0315	0.031*	
C36	0.1317 (3)	0.7596 (3)	0.06651 (18)	0.0252 (7)	
H36A	0.1286	0.8249	0.0253	0.030*	
H36B	0.0605	0.7732	0.0986	0.030*	
C37	0.1246 (4)	0.6381 (3)	0.04706 (17)	0.0281 (8)	
H37A	0.1869	0.6319	0.0088	0.034*	
H37B	0.0443	0.6354	0.0308	0.034*	
C41	0.0274 (3)	0.4071 (3)	0.11525 (17)	0.0230 (7)	
C42	−0.0919 (3)	0.4487 (3)	0.13059 (17)	0.0267 (7)	
H42A	−0.1088	0.5300	0.1356	0.032*	
C43	−0.1864 (3)	0.3721 (4)	0.13863 (19)	0.0322 (8)	
H43A	−0.2674	0.4003	0.1499	0.039*	
C44	−0.1616 (4)	0.2546 (4)	0.13016 (19)	0.0352 (9)	
H44A	−0.2257	0.2016	0.1365	0.042*	
C45	−0.0438 (4)	0.2132 (3)	0.1125 (2)	0.0353 (9)	
H45A	−0.0276	0.1330	0.1056	0.042*	
C46	0.0501 (4)	0.2903 (3)	0.10514 (19)	0.0287 (8)	
H46A	0.1307	0.2624	0.0930	0.034*	
C51	0.2878 (3)	0.4266 (3)	0.09389 (17)	0.0238 (7)	
C52	0.3191 (4)	0.4080 (4)	0.0279 (2)	0.0356 (9)	
H52A	0.2669	0.4395	−0.0067	0.043*	
C53	0.4262 (4)	0.3436 (4)	0.0130 (2)	0.0443 (10)	
H53A	0.4468	0.3311	−0.0319	0.053*	
C54	0.5031 (4)	0.2975 (4)	0.0622 (2)	0.0396 (10)	
H54A	0.5763	0.2534	0.0513	0.047*	
C55	0.4740 (4)	0.3153 (3)	0.1277 (2)	0.0335 (8)	
H55A	0.5274	0.2843	0.1617	0.040*	
C56	0.3655 (3)	0.3792 (3)	0.14365 (18)	0.0262 (7)	
H56A	0.3448	0.3903	0.1888	0.031*	
P10	0.78890 (8)	0.89315 (8)	0.08279 (5)	0.0250 (2)	
F11	0.7426 (2)	1.03165 (19)	0.07981 (13)	0.0408 (6)	
F12	0.8351 (2)	0.7545 (2)	0.08694 (13)	0.0425 (6)	
F13	0.6962 (2)	0.8529 (2)	0.14901 (11)	0.0391 (5)	
F14	0.6869 (2)	0.8796 (2)	0.03586 (12)	0.0380 (5)	
F15	0.8896 (2)	0.9069 (2)	0.13115 (12)	0.0383 (5)	
F16	0.8808 (2)	0.9332 (2)	0.01755 (12)	0.0446 (6)	
P20	0.49733 (11)	0.28058 (11)	0.35088 (6)	0.0413 (3)	
F21	0.5132 (3)	0.4160 (3)	0.35538 (17)	0.0684 (9)	
F22	0.6133 (3)	0.2418 (3)	0.38990 (17)	0.0776 (10)	
F23	0.5813 (3)	0.3003 (3)	0.28115 (14)	0.0584 (7)	
F24	0.3828 (3)	0.3256 (5)	0.31074 (19)	0.1052 (15)	
F25	0.4106 (3)	0.2649 (4)	0.42035 (17)	0.0807 (11)	
F26	0.4822 (5)	0.1461 (4)	0.3489 (3)	0.134 (2)	
N200	0.8799 (7)	0.0600 (8)	0.3056 (6)	0.149 (4)	
C201	0.7923 (8)	0.0335 (5)	0.2890 (4)	0.083 (2)	
C202	0.6818 (7)	0.0013 (7)	0.2705 (4)	0.095 (2)	
H20A	0.6639	−0.0804	0.2956	0.143*	
H20B	0.6895	0.0032	0.2215	0.143*	
H20C	0.6158	0.0589	0.2814	0.143*	
N300	0.6186 (8)	0.7019 (8)	0.3728 (4)	0.115 (4)	0.852 (11)
C301	0.5552 (7)	0.6772 (10)	0.4114 (4)	0.094 (3)	0.852 (11)
C302	0.4552 (18)	0.6296 (16)	0.4684 (10)	0.226 (9)*	0.852 (11)
H30A	0.4254	0.6941	0.4924	0.340*	0.852 (11)
H30B	0.4888	0.5606	0.5004	0.340*	0.852 (11)
H30C	0.3880	0.6041	0.4483	0.340*	0.852 (11)

### Atomic displacement parameters (Å^2^)

**Table:**

	*U*^11^	*U*^22^	*U*^33^	*U*^12^	*U*^13^	*U*^23^
Pd1	0.01780 (13)	0.02025 (13)	0.01547 (12)	−0.00351 (9)	−0.00124 (9)	−0.00340 (9)
P1	0.0217 (4)	0.0254 (4)	0.0219 (4)	−0.0036 (3)	−0.0001 (3)	−0.0083 (3)
P2	0.0215 (4)	0.0236 (4)	0.0166 (4)	−0.0023 (3)	−0.0028 (3)	−0.0046 (3)
N1	0.0255 (16)	0.0276 (14)	0.0201 (14)	−0.0059 (12)	−0.0026 (11)	−0.0044 (11)
N2	0.0201 (15)	0.0308 (15)	0.0194 (14)	−0.0036 (12)	−0.0037 (11)	−0.0046 (11)
N3	0.0177 (14)	0.0252 (14)	0.0219 (14)	−0.0021 (11)	−0.0013 (11)	−0.0048 (11)
N4	0.0154 (14)	0.0252 (14)	0.0220 (14)	−0.0003 (11)	−0.0003 (11)	−0.0018 (11)
C1	0.0240 (17)	0.0262 (16)	0.0161 (15)	−0.0055 (13)	−0.0041 (13)	−0.0026 (12)
C2	0.036 (2)	0.0240 (17)	0.033 (2)	−0.0044 (15)	0.0054 (16)	−0.0044 (15)
C3	0.040 (2)	0.040 (2)	0.041 (2)	0.0041 (18)	−0.0026 (19)	0.0013 (18)
C4	0.0293 (19)	0.0350 (19)	0.0197 (16)	−0.0143 (15)	−0.0004 (14)	−0.0025 (14)
C5	0.0194 (17)	0.0381 (19)	0.0191 (16)	−0.0084 (14)	−0.0014 (13)	−0.0029 (14)
C6	0.0195 (17)	0.0313 (18)	0.0280 (18)	0.0004 (14)	−0.0037 (14)	−0.0037 (14)
C7	0.0233 (19)	0.0316 (18)	0.037 (2)	0.0020 (14)	0.0004 (15)	−0.0144 (16)
C11	0.0280 (19)	0.0268 (17)	0.038 (2)	−0.0044 (15)	0.0049 (16)	−0.0161 (15)
C12	0.039 (2)	0.041 (2)	0.049 (3)	−0.0104 (19)	−0.0021 (19)	−0.0183 (19)
C13	0.051 (3)	0.045 (3)	0.085 (4)	−0.019 (2)	−0.001 (3)	−0.025 (3)
C14	0.055 (3)	0.029 (2)	0.103 (5)	−0.016 (2)	0.015 (3)	−0.020 (3)
C15	0.042 (3)	0.029 (2)	0.085 (4)	0.0002 (19)	0.010 (3)	0.000 (2)
C16	0.041 (2)	0.0262 (18)	0.048 (2)	−0.0008 (17)	0.0039 (19)	−0.0055 (17)
C21	0.0233 (18)	0.040 (2)	0.0210 (17)	−0.0080 (15)	−0.0005 (14)	−0.0111 (15)
C22	0.031 (2)	0.061 (3)	0.032 (2)	−0.0025 (19)	−0.0003 (17)	−0.021 (2)
C23	0.046 (3)	0.093 (4)	0.025 (2)	−0.013 (3)	0.0034 (19)	−0.025 (2)
C24	0.051 (3)	0.088 (4)	0.023 (2)	−0.019 (3)	−0.0053 (19)	−0.005 (2)
C25	0.054 (3)	0.056 (3)	0.037 (2)	−0.009 (2)	−0.014 (2)	0.001 (2)
C26	0.038 (2)	0.043 (2)	0.0251 (19)	−0.0060 (18)	−0.0043 (16)	−0.0071 (16)
C31	0.0152 (15)	0.0223 (15)	0.0224 (16)	0.0003 (12)	−0.0001 (12)	−0.0059 (13)
C32	0.029 (2)	0.0331 (18)	0.0267 (18)	−0.0038 (15)	−0.0117 (15)	−0.0027 (15)
C33	0.0221 (19)	0.035 (2)	0.041 (2)	−0.0003 (15)	−0.0095 (16)	−0.0027 (17)
C34	0.0209 (17)	0.0271 (17)	0.0292 (18)	−0.0048 (14)	0.0022 (14)	−0.0031 (14)
C35	0.0190 (17)	0.0252 (16)	0.0294 (18)	−0.0053 (13)	0.0014 (14)	0.0013 (14)
C36	0.0231 (18)	0.0273 (17)	0.0237 (17)	−0.0033 (14)	−0.0059 (14)	0.0006 (13)
C37	0.035 (2)	0.0307 (18)	0.0183 (16)	−0.0052 (15)	−0.0068 (14)	−0.0004 (14)
C41	0.0206 (17)	0.0292 (17)	0.0208 (16)	−0.0055 (13)	−0.0052 (13)	−0.0056 (13)
C42	0.0280 (19)	0.0326 (18)	0.0224 (17)	−0.0025 (15)	−0.0049 (14)	−0.0108 (14)
C43	0.0185 (18)	0.050 (2)	0.0310 (19)	−0.0053 (16)	−0.0038 (15)	−0.0132 (17)
C44	0.037 (2)	0.040 (2)	0.0292 (19)	−0.0187 (18)	−0.0048 (16)	−0.0021 (16)
C45	0.045 (2)	0.0256 (18)	0.036 (2)	−0.0063 (16)	−0.0165 (18)	−0.0016 (15)
C46	0.0286 (19)	0.0278 (17)	0.0307 (19)	0.0002 (14)	−0.0114 (15)	−0.0046 (14)
C51	0.0209 (17)	0.0302 (17)	0.0222 (16)	−0.0044 (14)	−0.0019 (13)	−0.0090 (14)
C52	0.033 (2)	0.050 (2)	0.0253 (19)	0.0013 (18)	−0.0042 (16)	−0.0124 (17)
C53	0.042 (3)	0.058 (3)	0.035 (2)	0.004 (2)	0.0067 (19)	−0.021 (2)
C54	0.023 (2)	0.050 (2)	0.047 (2)	0.0045 (17)	0.0025 (17)	−0.019 (2)
C55	0.0247 (19)	0.036 (2)	0.042 (2)	0.0003 (15)	−0.0078 (16)	−0.0120 (17)
C56	0.0204 (17)	0.0314 (18)	0.0274 (18)	−0.0026 (14)	−0.0020 (14)	−0.0076 (14)
P10	0.0195 (4)	0.0224 (4)	0.0318 (5)	−0.0031 (3)	−0.0055 (4)	−0.0006 (4)
F11	0.0470 (15)	0.0263 (11)	0.0493 (14)	0.0035 (10)	−0.0139 (12)	−0.0057 (10)
F12	0.0409 (14)	0.0265 (11)	0.0612 (16)	0.0034 (10)	−0.0146 (12)	−0.0085 (11)
F13	0.0283 (12)	0.0513 (14)	0.0342 (12)	−0.0092 (10)	0.0001 (10)	−0.0005 (10)
F14	0.0313 (12)	0.0439 (13)	0.0414 (13)	−0.0072 (10)	−0.0152 (10)	−0.0065 (10)
F15	0.0304 (12)	0.0381 (12)	0.0483 (14)	−0.0049 (10)	−0.0179 (10)	−0.0049 (10)
F16	0.0357 (14)	0.0526 (15)	0.0412 (14)	−0.0110 (11)	0.0058 (11)	−0.0023 (11)
P20	0.0336 (6)	0.0489 (6)	0.0426 (6)	−0.0133 (5)	−0.0027 (5)	−0.0088 (5)
F21	0.079 (2)	0.0559 (18)	0.070 (2)	−0.0091 (16)	0.0168 (17)	−0.0225 (16)
F22	0.060 (2)	0.099 (3)	0.061 (2)	0.0021 (18)	−0.0164 (16)	0.0151 (18)
F23	0.0574 (18)	0.0743 (19)	0.0451 (16)	−0.0043 (15)	0.0051 (13)	−0.0207 (14)
F24	0.045 (2)	0.204 (5)	0.070 (2)	−0.003 (2)	−0.0208 (17)	−0.031 (3)
F25	0.064 (2)	0.102 (3)	0.064 (2)	−0.0207 (19)	0.0251 (17)	0.0010 (19)
F26	0.142 (4)	0.080 (3)	0.194 (5)	−0.070 (3)	0.053 (4)	−0.070 (3)
N200	0.083 (5)	0.134 (7)	0.256 (12)	−0.020 (5)	−0.010 (6)	−0.094 (7)
C201	0.107 (6)	0.046 (3)	0.090 (5)	0.000 (4)	0.019 (4)	−0.018 (3)
C202	0.083 (5)	0.089 (5)	0.125 (7)	−0.010 (4)	0.006 (5)	−0.053 (5)
N300	0.104 (6)	0.121 (7)	0.088 (5)	0.024 (5)	0.051 (5)	0.008 (5)
C301	0.053 (5)	0.179 (10)	0.059 (5)	−0.013 (5)	0.005 (4)	−0.047 (6)

### Geometric parameters (Å, °)

**Table:**

Pd1—C31	2.031 (3)	C32—C33	1.504 (5)
Pd1—C1	2.033 (3)	C32—H32A	0.9900
Pd1—P1	2.3085 (9)	C32—H32B	0.9900
Pd1—P2	2.3100 (9)	C33—H33A	0.9800
P1—C21	1.806 (4)	C33—H33B	0.9800
P1—C11	1.811 (4)	C33—H33C	0.9800
P1—C7	1.848 (4)	C34—C35	1.344 (5)
P2—C51	1.805 (4)	C34—H34A	0.9500
P2—C41	1.815 (3)	C35—H35A	0.9500
P2—C37	1.847 (3)	C36—C37	1.523 (5)
N1—C1	1.347 (4)	C36—H36A	0.9900
N1—C4	1.387 (5)	C36—H36B	0.9900
N1—C2	1.467 (5)	C37—H37A	0.9900
N2—C1	1.347 (5)	C37—H37B	0.9900
N2—C5	1.379 (4)	C41—C46	1.383 (5)
N2—C6	1.466 (4)	C41—C42	1.395 (5)
N3—C31	1.348 (4)	C42—C43	1.391 (5)
N3—C34	1.386 (4)	C42—H42A	0.9500
N3—C32	1.467 (4)	C43—C44	1.381 (6)
N4—C31	1.347 (4)	C43—H43A	0.9500
N4—C35	1.387 (4)	C44—C45	1.389 (6)
N4—C36	1.459 (4)	C44—H44A	0.9500
C2—C3	1.512 (6)	C45—C46	1.391 (5)
C2—H2A	0.9900	C45—H45A	0.9500
C2—H2B	0.9900	C46—H46A	0.9500
C3—H3A	0.9800	C51—C56	1.391 (5)
C3—H3B	0.9800	C51—C52	1.400 (5)
C3—H3C	0.9800	C52—C53	1.383 (6)
C4—C5	1.339 (5)	C52—H52A	0.9500
C4—H4A	0.9500	C53—C54	1.375 (6)
C5—H5A	0.9500	C53—H53A	0.9500
C6—C7	1.528 (5)	C54—C55	1.382 (6)
C6—H6A	0.9900	C54—H54A	0.9500
C6—H6B	0.9900	C55—C56	1.397 (5)
C7—H7A	0.9900	C55—H55A	0.9500
C7—H7B	0.9900	C56—H56A	0.9500
C11—C12	1.386 (6)	P10—F16	1.584 (2)
C11—C16	1.392 (6)	P10—F14	1.595 (2)
C12—C13	1.384 (6)	P10—F12	1.601 (2)
C12—H12A	0.9500	P10—F13	1.603 (2)
C13—C14	1.362 (8)	P10—F11	1.603 (2)
C13—H13A	0.9500	P10—F15	1.605 (2)
C14—C15	1.371 (8)	P20—F26	1.558 (4)
C14—H14A	0.9500	P20—F22	1.569 (3)
C15—C16	1.396 (6)	P20—F24	1.577 (4)
C15—H15A	0.9500	P20—F21	1.583 (3)
C16—H16A	0.9500	P20—F23	1.586 (3)
C21—C22	1.391 (5)	P20—F25	1.598 (3)
C21—C26	1.392 (6)	N200—C201	1.147 (10)
C22—C23	1.390 (6)	C201—C202	1.421 (11)
C22—H22A	0.9500	C202—H20A	0.9800
C23—C24	1.359 (8)	C202—H20B	0.9800
C23—H23A	0.9500	C202—H20C	0.9800
C24—C25	1.378 (7)	N300—C301	1.000 (9)
C24—H24A	0.9500	C301—C302	1.549 (19)
C25—C26	1.391 (6)	C302—H30A	0.9800
C25—H25A	0.9500	C302—H30B	0.9800
C26—H26A	0.9500	C302—H30C	0.9800
			
C31—Pd1—C1	173.56 (13)	C33—C32—H32B	109.4
C31—Pd1—P1	94.24 (9)	H32A—C32—H32B	108.0
C1—Pd1—P1	84.03 (10)	C32—C33—H33A	109.5
C31—Pd1—P2	83.54 (9)	C32—C33—H33B	109.5
C1—Pd1—P2	96.46 (10)	H33A—C33—H33B	109.5
P1—Pd1—P2	164.44 (3)	C32—C33—H33C	109.5
C21—P1—C11	105.73 (18)	H33A—C33—H33C	109.5
C21—P1—C7	105.68 (18)	H33B—C33—H33C	109.5
C11—P1—C7	106.11 (17)	C35—C34—N3	107.0 (3)
C21—P1—Pd1	117.44 (13)	C35—C34—H34A	126.5
C11—P1—Pd1	115.02 (12)	N3—C34—H34A	126.5
C7—P1—Pd1	105.96 (12)	C34—C35—N4	106.4 (3)
C51—P2—C41	106.27 (16)	C34—C35—H35A	126.8
C51—P2—C37	107.06 (17)	N4—C35—H35A	126.8
C41—P2—C37	105.51 (16)	N4—C36—C37	111.9 (3)
C51—P2—Pd1	115.17 (11)	N4—C36—H36A	109.2
C41—P2—Pd1	115.95 (11)	C37—C36—H36A	109.2
C37—P2—Pd1	106.15 (12)	N4—C36—H36B	109.2
C1—N1—C4	110.3 (3)	C37—C36—H36B	109.2
C1—N1—C2	125.6 (3)	H36A—C36—H36B	107.9
C4—N1—C2	124.1 (3)	C36—C37—P2	114.2 (2)
C1—N2—C5	110.7 (3)	C36—C37—H37A	108.7
C1—N2—C6	123.4 (3)	P2—C37—H37A	108.7
C5—N2—C6	125.8 (3)	C36—C37—H37B	108.7
C31—N3—C34	110.5 (3)	P2—C37—H37B	108.7
C31—N3—C32	125.8 (3)	H37A—C37—H37B	107.6
C34—N3—C32	123.5 (3)	C46—C41—C42	119.2 (3)
C31—N4—C35	110.9 (3)	C46—C41—P2	123.0 (3)
C31—N4—C36	122.6 (3)	C42—C41—P2	117.6 (3)
C35—N4—C36	126.5 (3)	C43—C42—C41	120.5 (3)
N1—C1—N2	105.3 (3)	C43—C42—H42A	119.8
N1—C1—Pd1	133.2 (3)	C41—C42—H42A	119.8
N2—C1—Pd1	121.5 (2)	C44—C43—C42	119.5 (4)
N1—C2—C3	111.9 (3)	C44—C43—H43A	120.2
N1—C2—H2A	109.2	C42—C43—H43A	120.2
C3—C2—H2A	109.2	C43—C44—C45	120.7 (4)
N1—C2—H2B	109.2	C43—C44—H44A	119.7
C3—C2—H2B	109.2	C45—C44—H44A	119.7
H2A—C2—H2B	107.9	C44—C45—C46	119.3 (4)
C2—C3—H3A	109.5	C44—C45—H45A	120.3
C2—C3—H3B	109.5	C46—C45—H45A	120.3
H3A—C3—H3B	109.5	C41—C46—C45	120.8 (4)
C2—C3—H3C	109.5	C41—C46—H46A	119.6
H3A—C3—H3C	109.5	C45—C46—H46A	119.6
H3B—C3—H3C	109.5	C56—C51—C52	119.0 (3)
C5—C4—N1	106.9 (3)	C56—C51—P2	119.1 (3)
C5—C4—H4A	126.6	C52—C51—P2	121.9 (3)
N1—C4—H4A	126.6	C53—C52—C51	119.9 (4)
C4—C5—N2	106.9 (3)	C53—C52—H52A	120.0
C4—C5—H5A	126.6	C51—C52—H52A	120.0
N2—C5—H5A	126.6	C54—C53—C52	120.9 (4)
N2—C6—C7	112.6 (3)	C54—C53—H53A	119.5
N2—C6—H6A	109.1	C52—C53—H53A	119.5
C7—C6—H6A	109.1	C53—C54—C55	120.0 (4)
N2—C6—H6B	109.1	C53—C54—H54A	120.0
C7—C6—H6B	109.1	C55—C54—H54A	120.0
H6A—C6—H6B	107.8	C54—C55—C56	119.7 (4)
C6—C7—P1	114.3 (2)	C54—C55—H55A	120.1
C6—C7—H7A	108.7	C56—C55—H55A	120.1
P1—C7—H7A	108.7	C51—C56—C55	120.4 (3)
C6—C7—H7B	108.7	C51—C56—H56A	119.8
P1—C7—H7B	108.7	C55—C56—H56A	119.8
H7A—C7—H7B	107.6	F16—P10—F14	90.43 (14)
C12—C11—C16	119.9 (4)	F16—P10—F12	90.52 (14)
C12—C11—P1	121.1 (3)	F14—P10—F12	90.56 (13)
C16—C11—P1	118.5 (3)	F16—P10—F13	179.76 (15)
C13—C12—C11	119.8 (5)	F14—P10—F13	89.78 (13)
C13—C12—H12A	120.1	F12—P10—F13	89.59 (14)
C11—C12—H12A	120.1	F16—P10—F11	90.12 (14)
C14—C13—C12	120.4 (5)	F14—P10—F11	89.97 (13)
C14—C13—H13A	119.8	F12—P10—F11	179.17 (14)
C12—C13—H13A	119.8	F13—P10—F11	89.78 (14)
C13—C14—C15	120.4 (4)	F16—P10—F15	90.59 (14)
C13—C14—H14A	119.8	F14—P10—F15	178.91 (14)
C15—C14—H14A	119.8	F12—P10—F15	89.80 (13)
C14—C15—C16	120.5 (5)	F13—P10—F15	89.20 (13)
C14—C15—H15A	119.8	F11—P10—F15	89.66 (13)
C16—C15—H15A	119.8	F26—P20—F22	90.2 (3)
C11—C16—C15	118.9 (5)	F26—P20—F24	92.2 (3)
C11—C16—H16A	120.5	F22—P20—F24	177.4 (3)
C15—C16—H16A	120.5	F26—P20—F21	178.2 (3)
C22—C21—C26	119.7 (4)	F22—P20—F21	88.5 (2)
C22—C21—P1	120.1 (3)	F24—P20—F21	89.0 (2)
C26—C21—P1	120.2 (3)	F26—P20—F23	91.7 (2)
C23—C22—C21	119.9 (5)	F22—P20—F23	89.29 (18)
C23—C22—H22A	120.1	F24—P20—F23	89.78 (19)
C21—C22—H22A	120.1	F21—P20—F23	89.62 (17)
C24—C23—C22	119.8 (4)	F26—P20—F25	89.6 (2)
C24—C23—H23A	120.1	F22—P20—F25	91.9 (2)
C22—C23—H23A	120.1	F24—P20—F25	88.9 (2)
C23—C24—C25	121.4 (4)	F21—P20—F25	89.04 (19)
C23—C24—H24A	119.3	F23—P20—F25	178.2 (2)
C25—C24—H24A	119.3	N200—C201—C202	178.2 (9)
C24—C25—C26	119.5 (5)	C201—C202—H20A	109.5
C24—C25—H25A	120.2	C201—C202—H20B	109.5
C26—C25—H25A	120.2	H20A—C202—H20B	109.5
C25—C26—C21	119.6 (4)	C201—C202—H20C	109.5
C25—C26—H26A	120.2	H20A—C202—H20C	109.5
C21—C26—H26A	120.2	H20B—C202—H20C	109.5
N4—C31—N3	105.2 (3)	N300—C301—C302	175.1 (14)
N4—C31—Pd1	121.4 (2)	C301—C302—H30A	109.5
N3—C31—Pd1	133.0 (2)	C301—C302—H30B	109.5
N3—C32—C33	111.4 (3)	H30A—C302—H30B	109.5
N3—C32—H32A	109.4	C301—C302—H30C	109.5
C33—C32—H32A	109.4	H30A—C302—H30C	109.5
N3—C32—H32B	109.4	H30B—C302—H30C	109.5
			
C31—Pd1—P1—C21	−121.77 (16)	Pd1—P1—C21—C26	11.0 (4)
C1—Pd1—P1—C21	64.46 (16)	C26—C21—C22—C23	−0.6 (6)
P2—Pd1—P1—C21	157.10 (16)	P1—C21—C22—C23	−179.7 (3)
C31—Pd1—P1—C11	3.65 (18)	C21—C22—C23—C24	−0.6 (7)
C1—Pd1—P1—C11	−170.13 (18)	C22—C23—C24—C25	1.4 (8)
P2—Pd1—P1—C11	−77.49 (19)	C23—C24—C25—C26	−0.9 (7)
C31—Pd1—P1—C7	120.51 (16)	C24—C25—C26—C21	−0.3 (7)
C1—Pd1—P1—C7	−53.27 (16)	C22—C21—C26—C25	1.0 (6)
P2—Pd1—P1—C7	39.38 (19)	P1—C21—C26—C25	−179.9 (3)
C31—Pd1—P2—C51	67.33 (16)	C35—N4—C31—N3	−1.1 (4)
C1—Pd1—P2—C51	−119.15 (16)	C36—N4—C31—N3	178.9 (3)
P1—Pd1—P2—C51	149.91 (16)	C35—N4—C31—Pd1	173.0 (2)
C31—Pd1—P2—C41	−167.71 (16)	C36—N4—C31—Pd1	−7.0 (4)
C1—Pd1—P2—C41	5.81 (16)	C34—N3—C31—N4	1.2 (4)
P1—Pd1—P2—C41	−85.13 (17)	C32—N3—C31—N4	−173.9 (3)
C31—Pd1—P2—C37	−50.94 (16)	C34—N3—C31—Pd1	−171.9 (3)
C1—Pd1—P2—C37	122.58 (16)	C32—N3—C31—Pd1	13.0 (5)
P1—Pd1—P2—C37	31.64 (19)	P1—Pd1—C31—N4	−107.3 (3)
C4—N1—C1—N2	1.3 (4)	P2—Pd1—C31—N4	57.2 (3)
C2—N1—C1—N2	−179.1 (3)	P1—Pd1—C31—N3	64.8 (3)
C4—N1—C1—Pd1	−175.5 (3)	P2—Pd1—C31—N3	−130.6 (3)
C2—N1—C1—Pd1	4.1 (5)	C31—N3—C32—C33	108.9 (4)
C5—N2—C1—N1	−1.3 (4)	C34—N3—C32—C33	−65.6 (4)
C6—N2—C1—N1	−178.1 (3)	C31—N3—C34—C35	−0.8 (4)
C5—N2—C1—Pd1	175.9 (2)	C32—N3—C34—C35	174.4 (3)
C6—N2—C1—Pd1	−0.9 (4)	N3—C34—C35—N4	0.1 (4)
P1—Pd1—C1—N1	−130.2 (3)	C31—N4—C35—C34	0.6 (4)
P2—Pd1—C1—N1	65.4 (3)	C36—N4—C35—C34	−179.4 (3)
P1—Pd1—C1—N2	53.4 (3)	C31—N4—C36—C37	−63.7 (4)
P2—Pd1—C1—N2	−110.9 (3)	C35—N4—C36—C37	116.4 (4)
C1—N1—C2—C3	120.2 (4)	N4—C36—C37—P2	52.2 (4)
C4—N1—C2—C3	−60.2 (5)	C51—P2—C37—C36	−113.4 (3)
C1—N1—C4—C5	−0.8 (4)	C41—P2—C37—C36	133.7 (3)
C2—N1—C4—C5	179.6 (3)	Pd1—P2—C37—C36	10.2 (3)
N1—C4—C5—N2	−0.1 (4)	C51—P2—C41—C46	6.3 (3)
C1—N2—C5—C4	0.9 (4)	C37—P2—C41—C46	119.8 (3)
C6—N2—C5—C4	177.6 (3)	Pd1—P2—C41—C46	−123.1 (3)
C1—N2—C6—C7	−65.3 (4)	C51—P2—C41—C42	−179.2 (3)
C5—N2—C6—C7	118.4 (4)	C37—P2—C41—C42	−65.8 (3)
N2—C6—C7—P1	46.9 (4)	Pd1—P2—C41—C42	51.4 (3)
C21—P1—C7—C6	−109.5 (3)	C46—C41—C42—C43	2.8 (5)
C11—P1—C7—C6	138.5 (3)	P2—C41—C42—C43	−171.8 (3)
Pd1—P1—C7—C6	15.8 (3)	C41—C42—C43—C44	−1.1 (5)
C21—P1—C11—C12	20.4 (4)	C42—C43—C44—C45	−1.2 (6)
C7—P1—C11—C12	132.4 (3)	C43—C44—C45—C46	1.6 (6)
Pd1—P1—C11—C12	−110.9 (3)	C42—C41—C46—C45	−2.4 (5)
C21—P1—C11—C16	−167.6 (3)	P2—C41—C46—C45	172.0 (3)
C7—P1—C11—C16	−55.7 (3)	C44—C45—C46—C41	0.2 (6)
Pd1—P1—C11—C16	61.1 (3)	C41—P2—C51—C56	−107.9 (3)
C16—C11—C12—C13	0.8 (6)	C37—P2—C51—C56	139.7 (3)
P1—C11—C12—C13	172.6 (4)	Pd1—P2—C51—C56	21.9 (3)
C11—C12—C13—C14	−1.8 (7)	C41—P2—C51—C52	69.9 (3)
C12—C13—C14—C15	1.4 (8)	C37—P2—C51—C52	−42.5 (4)
C13—C14—C15—C16	0.0 (8)	Pd1—P2—C51—C52	−160.2 (3)
C12—C11—C16—C15	0.6 (6)	C56—C51—C52—C53	−0.2 (6)
P1—C11—C16—C15	−171.4 (3)	P2—C51—C52—C53	−178.1 (3)
C14—C15—C16—C11	−1.0 (7)	C51—C52—C53—C54	−0.2 (7)
C11—P1—C21—C22	60.2 (3)	C52—C53—C54—C55	−0.1 (7)
C7—P1—C21—C22	−52.0 (3)	C53—C54—C55—C56	0.7 (6)
Pd1—P1—C21—C22	−169.9 (3)	C52—C51—C56—C55	0.8 (5)
C11—P1—C21—C26	−118.9 (3)	P2—C51—C56—C55	178.8 (3)
C7—P1—C21—C26	128.9 (3)	C54—C55—C56—C51	−1.1 (6)
